# Evaluation of dopamine D_3_ receptor occupancy by blonanserin using [^11^C]-(+)-PHNO in schizophrenia patients

**DOI:** 10.1007/s00213-020-05698-3

**Published:** 2020-11-12

**Authors:** Takeshi Sakayori, Amane Tateno, Ryosuke Arakawa, Woo-chan Kim, Yoshiro Okubo

**Affiliations:** Department of Neuropsychiatry, Nippon Medical School, 1-1-5 Sendagi, Bunkyo-ku, Tokyo, 113–8602 Japan

**Keywords:** D_3_ receptor, Schizophrenia, Blonanserin, Positron emission tomography

## Abstract

**Rationale:**

Unlike other antipsychotics, our previous positron emission tomography (PET) study demonstrated that a single dose of blonanserin occupied dopamine D_3_ as well as dopamine D_2_ receptors in healthy subjects. However, there has been no study concerning the continued use of blonanserin.

**Objectives:**

We examined D_2_ and D_3_ receptor occupancies in patients with schizophrenia who had been treated with blonanserin.

**Methods:**

Thirteen patients with schizophrenia participated. PET examinations were performed on patients treated with clinical dosage of blonanserin or olanzapine alone. A crossover design was used in which seven patients switched drugs after the first scan, and PET examinations were conducted again. D_2_ and D_3_ receptor occupancies were evaluated by [^11^C]-(+)-PHNO. We used nondisplaceable binding potential (BP_ND_) of 6 healthy subjects which we previously reported as baseline. To consider the effect of upregulation of D_3_ receptor by continued use of antipsychotics, D_3_ receptor occupancy by blonanserin in seven subjects who completed 2 PET scans were re-analyzed by using BP_ND_ of olanzapine condition as baseline.

**Results:**

Average occupancy by olanzapine (10.8 ± 6.0 mg/day) was as follows: caudate 32.8 ± 18.3%, putamen 26.3 ± 18.2%, globus pallidus − 33.7 ± 34.9%, substantia nigra − 112.8 ± 90.7%. Average occupancy by blonanserin (12.8 ± 5.6 mg/day) was as follows: caudate 61.0 ± 8.3%, putamen 55.5 ± 9.5%, globus pallidus 48.9 ± 12.4%, substantia nigra 34.0 ± 20.6%. EC_50_ was 0.30 ng/mL for D_2_ receptor for caudate and putamen (df = 19, *p* < 0.0001) and 0.70 ng/mL for D_3_ receptor for globus pallidus and substantia nigra (df = 19, *p* < 0.0001). EC_50_ for D_3_ receptor of blonanserin changed to 0.22 ng/mL (df = 13, *p* = 0.0041) when we used BP_ND_ of olanzapine condition as baseline.

**Conclusions:**

Our study confirmed that blonanserin occupied both D_2_ and D_3_ receptors in patients with schizophrenia.

## Introduction

The dopamine D_2_ receptor family contains 3 subtypes (D_2_, D_3_, and D_4_), and they are known as the D_2_-like receptor family. In terms of the treatment of schizophrenia, D_2_ receptor has been thought to be strongly associated with the pathology of schizophrenia and be a major target for its treatment. Dopamine D_3_ receptor has similarities to the other members of the D_2_-like receptor family, but D_3_ receptor has very high affinity for dopamine and modulates dopamine release as an autoreceptor (Gross and Drescher 2012). Dopamine D_3_ receptors are predominantly located in the ventral striatum, thalamus, and hippocampus, which are important for psychotic symptoms and are thought to modulate normal dopaminergic function and cognition (Maramai et al. 2016). The results from PET studies with [^11^C]-(+)-PHNO indicated that 100% of the signal in the substantia nigra (SN), 67% in the globus pallidus (GP), and 26% in the ventral striatum represent D_3_ receptor sites (Searle et al. 2010). The distribution of D_3_ receptor in the limbic areas indicated that D_3_ receptor might regulate motivation and reward-related behavior (Leggio et al. 2013).

Selective D_3_ receptor antagonists affect the firing of dopaminergic neurons in the ventral striatum in a manner similar to atypical antipsychotics, and they enhance dopamine and acetylcholine release in the prefrontal cortex (Millan et al. 2008). It has also been indicated that D_3_ receptor antagonists can inhibit extrapyramidal symptoms and produce neither anhedonia nor metabolic adverse effects, mainly based on evidence from rodent studies (Richtand 2006; Young et al. 2012). D_3_ receptor antagonists can improve a series of social and cognitive behaviors in rodents, including executive functions, which are particularly impaired in patients with schizophrenia, while D_2_ antagonists do not have this effect (Gross et al. 2013). Since dopaminergic hypofunction in the prefrontal cortex has been implicated in the pathogenesis of negative symptoms (Davis et al. 1991) and cognitive dysfunctions of schizophrenia (Sawaguchi 2000), these findings led to the theoretical treatment model of D_3_ receptor antagonism being a valuable approach for the treatment of schizophrenia (Maramai et al. 2016). Thus, it seems worth verifying whether D_3_ receptor antagonism can improve the negative symptoms and cognitive deficits of schizophrenia.

Many antipsychotics have been reported with Ki values for D_2_ receptors not differing much from those for D_3_ receptors (McCormick et al. 2013), but occupancy of D_3_ receptors is moderately less than that of D_2_ receptors. It has been reported by a positron emission tomography (PET) study with [^11^C]-(+)-PHNO that several antipsychotics (i.e., clozapine, risperidone, olanzapine) did not decrease, or even increased the in vivo nondisplaceable binding potential (BP_ND_) of D_3_ receptors in human brain (Graff-Guerrero et al. 2009). These findings suggested that these antipsychotics hardly occupied D_3_ receptors in a clinical setting. [^11^C]-(+)-PHNO gives a mixed D_2/3_ signal composed of differing D_2_ and D_3_ proportions, and therefore previous studies measured BP_ND_ of D_3_ receptors in D_3_-receptor rich-regions. Another study also reported that chronically administered antipsychotics (i.e., clozapine, olanzapine, haloperidol) showed lower selectivity for D_3_ compared with D_2_ receptors ex vivo than in vitro in rat brain (McCormick et al. 2010).

Blonanserin is a second-generation antipsychotic drug developed in Japan, and it is currently being used as a therapeutic agent for schizophrenia in Japan, South Korea, and China. Comparative studies with other antipsychotic drugs have also been carried out, suggesting the possibility of this drug contributing to the improvement of cognitive impairments and negative symptoms of mental disorders (Murasaki 2016; Kishi et al. 2019). Blonanserin reportedly occupied a D_3_-rich region (i.e., cerebellum lobes 9–10) similarly to a D_2_-rich region (i.e., striatum) in rat brain, while risperidone, olanzapine, and aripiprazole did not (Baba et al. 2015). We recently examined the occupancy of D_2_ and D_3_ receptors by blonanserin in healthy subjects (Tateno et al. 2018). Using [^11^C]-(+)-PHNO and PET, we demonstrated that a single dose of 12 mg of blonanserin occupied D_3_ receptors to the same degree as D_2_ receptors (i.e., EC_50_ for the D_2_-rich region was 0.39 ng/mL and for the D_3_-rich region was 0.40 ng/mL) (Tateno et al. 2018). This finding led us to suggest the possibility that some of the pharmacological effect of blonanserin in schizophrenia patients might be mediated via D_3_ receptor antagonism. However, the result from the single-dose administration of blonanserin in healthy subjects may not reflect actual clinical practices as patients obtained antipsychotic effects by its continuous administration. Therefore, it is important to confirm how the continued use of blonanserin occupied D_3_ receptor of patients with schizophrenia in a clinical setting.

We hypothesized that blonanserin would occupy D_3_ receptor to the same degree as D_2_ receptor in patients with schizophrenia, in a manner similar to healthy subjects. In the present study, we evaluated both D_2_ and D_3_ receptor occupancy by blonanserin in patients with schizophrenia and compared the results with those by olanzapine, which has been demonstrated as not occupying D_3_ receptor (Mizrahi et al. 2011).

## Methods

### Subjects and study design

We selected a group of patients, aged 20 to 70 years, who met the criteria of Diagnostic and Statistical Manual of Mental Disorders (DSM)-IV for schizophrenia. Inclusion criteria were as follows: (1) schizophrenia patients treated with blonanserin or olanzapine alone for 4 weeks or more, and those who did not change their dose for at least 2 weeks; (2) subjects who agreed to change from one drug to the other, (3) subjects who scored less than 120 on the Positive and Negative Syndrome Scale (PANSS15) at screening. Exclusion criteria were as follows: (1) subjects with past or current serious medical illness and/or organic brain diseases, (2) subjects with contraindication for the use of magnetic resonance imaging (MRI), (3) subjects with contraindication for blonanserin and olanzapine, (4) subjects treated with electroconvulsive therapy within 3 months before the screening, (5) subjects taking tandospirone at the time of screening, as there was a report that buspirone, which is in the same drug family, occupies D_3_ receptor (Le Foll et al. 2016), and (6) subjects who were judged to be unsuitable for participation in this study. We allowed concomitant drugs such as benzodiazepines, antihypertensive drugs, and antiparkinsonian drugs that do not act on dopamine.

This study was designed as an open-label protocol. PET examination was performed on subjects who had been treated with blonanserin or olanzapine. A crossover design was then used in which patients switched drugs after the first scan, and PET examination was performed again after 2 weeks or longer. The doses of both drugs were within their clinical dose range. The mean dose of olanzapine was 10.8 ± 6.0 (range: 2.5–20) mg/day, and that of blonanserin was 12.8 ± 5.6 (8–24) mg/day. After complete explanation of the study, written informed consent was obtained from all participants. This study was approved by the institutional review board of Nippon Medical School Hospital, Japan.

Thirteen subjects participated in this study. The patients’ characteristics are listed in Table 1. None of them worsened greatly after the medication change. Seven patients completed 2 PET scans, five of whom took olanzapine first and two were given blonanserin first. Six subjects were examined only by the 1st PET scan, 3 of whom participated in the PET scan with only blonanserin, and 3 with only olanzapine. Three of them felt uneasy about the drug change after the 1st PET scan and decided to continue with the initial drug, and the other 3 discontinued because of their clinical condition before the 2nd PET scan; one was diagnosed with diabetes , one complained of insomnia, and one was stopped due to extrapyramidal symptoms.

**Table 1:** Patient characteristics, dose, plasma concentration, and binding potential (BP_ND_) of each ROI by olanzapine and blonanserin. *CAU*, caudate; *PUT*, putamen; *GP*, globus pallidus; *SN*, substantia nigra

ID	Gender	Age (years)	PANSS total	Olanzapine						Blonanserin					
				Dose (mg/day)	Plasma concentration (ng/mL)	Binding potential				Dose (mg/day)	Plasma concentration (ng/mL)	Binding potential			
						CAU	PUT	GP	SN			CAU	PUT	GP	SN
*01*	Male	62	76	*20*	67.3	0.77	1.09	2.62	2.38	*24*	0.851	0.42	0.62	0.76	0.44
*02*	Male	66	76	*10*	31.7	1.07	1.41	2.93	2.07	*N/A*	N/A	N/A	N/A	N/A	N/A
*03*	Female	69	53	*5*	21.9	0.93	1.17	2.35	1.82	*8*	0.414	0.58	0.77	1.11	0.60
*04*	Female	57	67	*20*	45.6	0.76	1.04	2.82	1.56	*N/A*	N/A	N/A	N/A	N/A	N/A
*05*	Male	46	62	*10*	29.4	0.98	1.30	3.34	4.31	*16*	0.518	0.71	0.92	1.24	0.86
*06*	Female	37	63	*5*	18.4	1.35	1.70	4.34	2.45	*8*	0.096	0.77	1.03	1.57	0.86
*07*	Female	46	72	*10*	50.7	1.30	1.72	3.83	3.43	*16*	0.856	0.62	0.90	0.86	0.56
*08*	Male	42	64	*N/A*	N/A	N/A	N/A	N/A	N/A	*8*	0.234	0.68	0.91	1.11	0.94
*09*	Male	55	53	*2.5*	13	1.02	1.31	2.38	1.73	*8*	1.08	0.69	0.88	1.27	0.80
*10*	Female	50	62	*15*	0.0161	1.52	1.84	2.33	0.98	*N/A*	N/A	N/A	N/A	N/A	N/A
*11*	Male	24	77	*10*	67.2	0.66	0.82	2.39	1.92	*16*	0.848	0.45	0.50	0.85	0.37
*12*	Female	47	84	*N/A*	N/A	N/A	N/A	N/A	N/A	*16*	0.421	0.42	0.64	0.89	0.59
*13*	Male	24	64	*N/A*	N/A	N/A	N/A	N/A	N/A	*8*	0.241	0.64	0.94	1.40	1.02
Average SD		48.1 14.2	67.2 9.4	10.8 6.0	34.5 22.7	1.04 0.28	1.34 0.33	2.93 0.70	2.27 0.96	12.8 5.6	0.56 0.33	0.60 0.13	0.81 0.17	1.11 0.27	0.70 0.22

### PET procedures

PET scans were performed with Eminence SET-3000GCT-X (Shimadzu Corp, Kyoto, Japan) to measure regional brain radioactivity. This scanner provides 99 sections with an axial field of view of 26.0 cm. Spatial resolution was 3.45 mm in-plane and 3.72 mm axially full-width at half-maximum. A head fixation device was used during the scans. A 15-min transmission scan was done to correct for attenuation using a ^137^Cs source. Dynamic PET scan was performed for 90 min (1 min × 15, 5 min × 15) after i.v. bolus injection of [^11^C]-(+)-PHNO. Injected radioactivity was 139.1 to 386.4 MBq (309.8 ± 79.7 (mean ± SD MBq) for olanzapine-condition; 351.2 ± 34.3 MBq for blonanserin-condition). The injected mass of [^11^C]-(+)-PHNO was 0.5–2.5 μg (2.0 ± 0.7 μg for olanzapine-condition; 2.4 ± 0.3 μg for blonanserin-condition). Molar radioactivity was 55.3–141.0 GBq/μmol (86.0 ± 25.7 GBq/μmol for olanzapine-condition; 77.5 ± 21.9 GBq/μmol for blonanserin -condition) at the time of injection.

### MRI procedures

MRI of the brain was acquired with 1.5 T MR imaging, Intera 1.5 T Achieva Nova (Philips Medical Systems, Best, Netherlands) as proton density image (echo time = 17 ms; repetition time = 6000 ms; field of view = 22 cm, 2-dimensional, 256 × 256; slice thickness = 2 mm; number of excitations = 2). These images were used for analysis of the PET scans.

### Measurement of plasma concentrations of blonanserin and olanzapine

Venous blood samples were taken just before the PET scans, collected in tubes containing EDTA-2Na, and centrifuged at 3000 rpm for 10 min at 4 °C. Separated plasma samples were stored at − 80 °C until analysis. The plasma concentration of blonanserin was measured by validated method using high-performance liquid chromatography-tandem mass spectrometry with a target lower quantification limit of 0.001 ng/mL (Sekisui Medical Co., Ltd., Tokyo, Japan). The plasma concentration of olanzapine was measured by validated method using high-performance liquid chromatography-tandem mass spectrometry with a target lower quantification limit of 0.0001 ng/mL (Sumika Chemical Analysis Service Co., Ltd., Osaka, Japan).

### PET data analysis

MR images were co-registered to summated PET images with the mutual information algorithm using PMOD (version 3.4; PMOD Technologies Ltd., Zurich, Switzerland). Regions of interest (ROIs) were defined for the caudate, putamen, globus pallidus, substantia nigra, and cerebellum in accordance with Tziortzi’s study (Tziortzi et al. 2011). We defined the caudate and putamen as D_2_-rich regions and the substantia nigra and globus pallidus as D_3_-rich regions, based on Searle’s study with [^11^C]-(+)-PHNO (Searle et al. 2010). ROIs were drawn manually on overlaid summated PET and co-registered MR images of each subject. By matching the targeted frame to the average of the first 10 frames (i.e., 0–10 min), motion corrections were conducted in all subjects.

Quantitative estimate of binding of [^11^C]-(+)-PHNO was performed using a simplified reference tissue model (Lammertsma and Hume 1996), with the cerebellar cortex as reference region. We avoided cerebellum midline-structures because of measurable specific [^11^C]-(+)-PHNO binding. This model has been validated to reliably estimate BP_ND_, which compares the concentration of radioligand in the receptor-rich region with the receptor-free region (Innis et al. 2007) for [^11^C]-(+)-PHNO (Ginovart et al. 2007).

Receptor occupancy by drugs was calculated by the following equation:

$$ \mathrm{Occupancy}\ \left(\%\right)=\left({\mathrm{BP}}_{\mathrm{NDbase}}-{\mathrm{BP}}_{\mathrm{NDdrug}}\right)/{\mathrm{BP}}_{\mathrm{NDbase}}\times 100 $$

BP_NDdrug_ is the BP_ND_ of schizophrenia patients treated with blonanserin or olanzapine. The BP_ND_ value of 6 healthy male volunteers (HVs) (age range 27–46 years; mean ± SD, 35.7 ± 7.6), which we reported in a previous study (Tateno et al. 2018), was used as baseline (BP_NDbase_) (Table 2). Average BP_ND_ in the healthy volunteers under drug-free condition was as follows: caudate (range 1.04–1.68; mean ± SD 1.53 ± 0.24), putamen (1.28–2.06; 1.82 ± 0.29), globus pallidus (1.56–2.68; 2.16 ± 0.40), and substantia nigra (0.96–1.42; 1.06 ± 0.17).

**Table 2:** BP_ND_ values for the healthy volunteers in each region (Tateno et al. 2018). *HV*, healthy volunteers; *CAU*, caudate; *PUT*, putamen; *GP*, globus pallidus; *SN*, substantia nigra

ID	Gender	Age (years)	Drug-free			
			Binding potential			
			CAU	PUT	GP	SN
HV-1	Male	42	1.58	1.71	2.04	1.03
HV-2	Male	46	1.68	1.93	1.98	0.98
HV-3	Male	29	1.59	1.86	2.22	0.96
HV-4	Male	32	1.67	2.04	2.68	1.42
HV-5	Male	38	1.64	2.06	2.50	0.99
HV-6	Male	27	1.04	1.28	1.56	1.01
Average SD		35.7 7.6	1.53 0.24	1.82 0.29	2.16 0.40	1.06 0.17

We used a 1-site binding model, the same as in a previous study (Graff-Guerrero et al. 2010). The relationship between plasma concentration and receptor occupancy was shown by the following equation:

$$ \mathrm{Occupancy}\ \left(\%\right)={E}_{\mathrm{max}}\times C/\left({\mathrm{EC}}_{50}+C\right)\times 100, $$

where *C* is the plasma concentration of drug, *E*_max_ is the maximum occupancy, and EC_50_ is the plasma concentration required to achieve 50% occupancy (Tateno et al. 2018; Graff-Guerrero et al. 2010). *E*_max_ was fixed at 1 and EC_50_ > 0, the same as in the previous occupancy studies (Tateno et al. 2018; Graff-Guerrero et al. 2010).

Mizrahi et al. reported that continuous intake of atypical antipsychotic drugs upregulated D_3_ receptors (Mizrahi et al. 2011). Upregulation of D_3_ receptors in treated schizophrenia patients might increase BP_ND_, which induces the underestimation of occupancy of antipsychotics when using HV as baseline. To accurately compare D_3_ receptor occupancy with D_2_ receptor occupancy by blonanserin in consideration of the effect of the upregulation of D_3_ receptors, we also calculated the D_3_ receptor occupancy of blonanserin using individual BP_ND_ of olanzapine as a baseline among 7 patients who were taking both blonanserin and olanzapine. The paired *t* test was used to statistically analyze the comparison between D_3_ receptor occupancy of blonanserin by using BP_ND_ of olanzapine as baseline and that of healthy control as baseline.

## Results

The BP_ND_ values of each of the ROIs by olanzapine and blonanserin are summarized in Table 1.

### D_2_ and D_3_ receptor occupancies by olanzapine and blonanserin

**Fig. 1:**
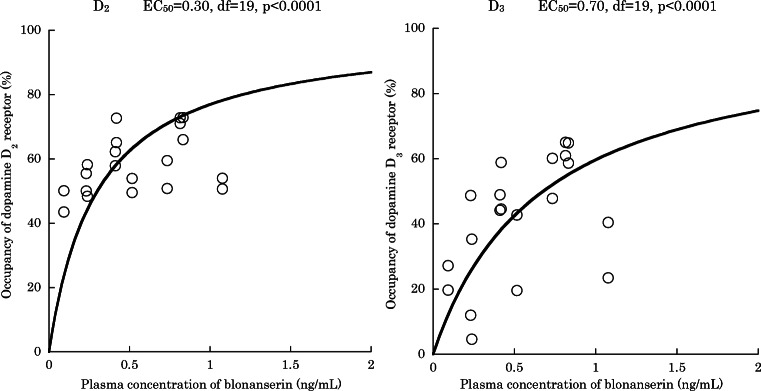
Correlation diagram of blonanserin plasma concentration and dopamine D_2_ (caudate nucleus and putamen) and D_3_ (globus pallidus and substantia nigra) receptor occupancy (*N* = 10). Baseline BP_ND_ was the average value of healthy volunteers

We analyzed D_2_ and D_3_ receptor occupancy using BP_ND_ of HV as baseline. The average occupancy by olanzapine (average ± SD, 10.8 ± 6.0 mg/day) was as follows: caudate nucleus 32.8 ± 18.3%, putamen 26.3 ± 18.2%, globus pallidus − 33.7 ± 34.9%, substantia nigra − 112.8 ± 90.7%. The average level of occupancy by blonanserin (12.8 ± 5.6 mg/day) was as follows: caudate nucleus 61.0 ± 8.3%, putamen 55.5 ± 9.5%, globus pallidus 48.9 ± 12.4%, substantia nigra 34.0 ± 20.6%. Correlations between the plasma concentration of blonanserin and receptor occupancy in D_2_-rich and D_3_-rich regions are shown in Fig. 1. EC_50_ of D_2_ receptor was 0.30 ng/mL (df = 19, *p* < 0.0001, 95% CI [0.215–0.394]), while EC_50_ of D_3_ receptor was 0.70 ng/mL (df = 19, *p* < 0.0001, 95% CI [0.478–0.919]).

### D_3_ receptor occupancy by blonanserin using individual BP_ND_ of olanzapine as baseline

**Fig. 2:**
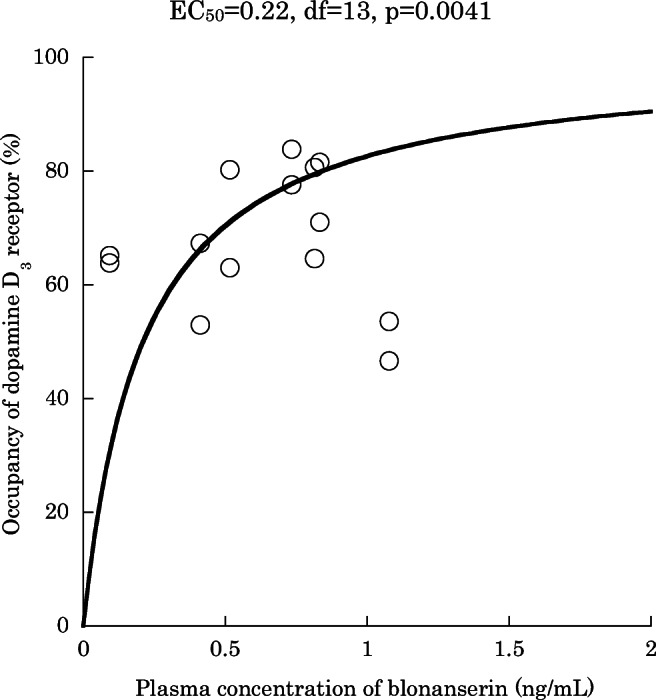
Correlation diagram of blonanserin plasma concentration and D_3_ (globus pallidus and substantia nigra) receptor occupancy (*N* = 7). Baseline BP_ND_ was the individual value under olanzapine condition

We also calculated the D_3_ receptor occupancy by blonanserin using individual BP_ND_ of olanzapine condition as baseline. The results are shown in Table 3. The occupancy of D_3_ was higher than when using the baseline BP_ND_ in healthy volunteers (67.9 ± 11.8% versus 44.7 ± 16.6%) (df = 26, *p* = 0.0002). EC_50_ of D_3_ receptor occupancy was 0.22 ng/mL (df = 13, *p* = 0.0041, 95% CI [0.095–0.341]), which was close to that of D_2_ receptor occupancy (Fig. 2).

**Table 3:** Dopamine D_2_ receptor and D_3_ receptor occupancy by blonanserin in seven patients who completed 2 PET scans. Baseline BP_ND_ was the average value of healthy volunteers for D_2_ receptor and the individual value at olanzapine condition for D_3_ receptor. *CAU*, caudate; *PUT*, putamen; *GP*, globus pallidus; *SN*, substantia nigra

ID	Occupancy of D_2_-rich region (%)		Occupancy of D_3_-rich region (%)	
	Baseline BP_ND_: average value of healthy volunteers		Baseline BP_ND_: individual value at olanzapine condition	
	CAU	PUT	GP	SN
01	72.8	65.9	70.9	81.4
03	62.1	57.8	52.9	67.2
05	53.8	49.5	62.9	80.1
06	50.0	43.4	63.8	65.1
07	59.4	50.7	77.5	83.8
09	53.9	50.6	46.7	53.7
11	70.9	72.7	64.5	80.6
Average SD	60.4 8.8	55.8 10.3	62.7 10.4	73.1 11.3

## Discussion

In this study, we confirmed that blonanserin indeed occupied D_3_ receptors in the globus pallidus and substantia nigra, although to a lesser degree than D_2_ receptors in the caudate nucleus and putamen, in patients with schizophrenia. On the other hand, olanzapine occupied 30% of the evaluation sites of D_2_ receptor, but hardly those of D_3_ receptor. These findings were consistent with a previous animal study and an in vivo human study (Baba et al. 2015; Graff-Guerrero et al. 2009).

The occupancy of D_3_ receptor by blonanserin was a little lower than in a previous study of healthy volunteers (Tateno et al. 2018), but it was similar when recalculated using individual BP_ND_ under olanzapine condition. First, upregulation of D_3_ receptors in treated schizophrenia patients with antipsychotics has been thought to influence BP_ND_ and the occupancy of antipsychotics (Graff-Guerrero et al. 2009; Mizrahi et al. 2011), and it might decrease the apparent D_3_ receptor occupancy. Regarding the upregulation by antipsychotics, it was earlier reported that occupancy of the globus pallidus by clozapine, olanzapine, and risperidon was − 70.7 ± 86.5% (Graff-Guerrero et al. 2009). Another study reported that occupancy of the globus pallidus by olanzapine and risperidone was − 50.28 ± 29.37% (Mizrahi et al. 2011). In the current study, we also used BP_ND_ of D_3_ receptors of patients under olanzapine treatment as a baseline for the calculation of D_3_ receptor occupancy to reduce the influence of upregulation. Although it was expected to show a similar value to those of previous studies, our result was that the D_3_ receptor occupancy by blonanserin (34.0 to 48.8%) was slightly lower than the D_2_ receptor occupancy (55.5 to 61.0%) using HV as baseline. This result seemed to be due to the influence of upregulation. Second, schizophrenia patients showed increased [^11^C]-(+)-PHNO binding compared to healthy subjects even if they were untreated (Weidenauer et al. 2020). For these reasons, individual baseline values would be more desirable. EC_50_ of D_3_ receptor by blonanserin changed from 0.70 to 0.22 ng/mL when switching baseline BP_ND_ from the average of healthy volunteers to the individual patient’s value with olanzapine in the 7 patients who had completed the 2 PET scans. This study assumes that the degree of upregulation was similar with olanzapine and blonanserin. This value was lower than EC_50_ of D_2_ receptor (0.40 ng/mL) for blonanserin in the same 7 subjects by using HV as baseline. Thus, our results confirmed that blonanserin occupied D_3_ receptor as well as D_2_ receptor in patients with schizophrenia. Blonanserin might be an important target for further studies regarding the therapeutic efficacy of D_3_ receptor blockade by antipsychotic drugs.

In this study, the degree of D_3_ receptor occupancy in the substantia nigra by olanzapine in 8 patients with schizophrenia was − 124.1 ± 87.6%. We thought that the negative occupancy might reflect upregulation. Previous studies did not measure the occupancy of the substantia nigra (Graff-Guerrero et al. 2009) or reported the combination of olanzapine (only one subject was included) and risperidone (Mizrahi et al. 2011). To our knowledge, this is the first report regarding the upregulation of dopamine D_3_ receptor by olanzapine; however, the evaluation of a large number of subjects and/or using same subjects both before and after its administration will be needed for clarification.

We should acknowledge several limitations to this study. First, our sample size was small. Furthermore, 6 of the 13 subjects underwent only one PET scan. Therefore, additional studies including larger numbers of subjects and longitudinal designs are essential for the generalization of our findings. Second, we used a younger-aged control group compared to the patients. BP_ND_ of D_2_ receptor was negatively correlated with age in the caudate, while that of D_3_ receptor was not correlated with age in the globus pallidus and substantia nigra (Nakajima et al. 2015). Our results of D_2_ receptor using younger-aged controls for baseline might be influenced by an age effect if controls would be older. Third, we used all-male HVs, whereas 46% of the patients were female. In this regard, a previous study indicated that D_3_ receptor differed between male and female rhesus monkeys (Martelle et al. 2014). Fourth, it is uncertain whether the degree of upregulation was similar or not between olanzapine and blonanserin. This study assumes that the degrees were comparable, although we could not estimate D_3_ upregulation exactly as there was no drug-free baseline condition. Fifth, 7 of the 13 participants in this study were smokers while all HVs were non-smokers. We could not rule out the effects of smoking, as it has been shown to have an effect on the dopamine system (Le Foll et al. 2014),

In conclusion, our study confirmed that continuous usage of blonanserin occupied dopamine D_3_ receptors to the same degree as D_2_ receptors in the brains of schizophrenia patients. By more discussions on the therapeutic effects of blonanserin, which is now known to clearly possess in vivo D_3_ receptor antagonism, we may be able to consider the relevance of anti-dopamine D_3_ receptor acivities as well as the therapeutic effects on cognitive impairments and negative symptoms of mental disorders.
